# Peptidomics Analysis of Virulent Peptides Involved in *Streptococcus suis* Pathogenesis

**DOI:** 10.3390/ani11092480

**Published:** 2021-08-24

**Authors:** Chadaporn Chaiden, Janthima Jaresitthikunchai, Narumon Phaonakrop, Sittiruk Roytrakul, Anusak Kerdsin, Suphachai Nuanualsuwan

**Affiliations:** 1 Department of Veterinary Public Health, Faculty of Veterinary Sciences, Chulalongkorn University, Bangkok 10330, Thailand; tiny_my_mild@outlook.com; 2 Functional Proteomics Technology Laboratory, Functional Ingredients and Food Innovation Research Group, National Center for Genetic Engineering and Biotechnology, National Science and Technology for Development Agency, Pathum Thani 12120, Thailand; janthima.jar@biotec.or.th (J.J.); narumon.pha@biotec.or.th (N.P.); 3 Faculty of Public Health, Kasetsart University Chalermphrakiat Sakon Nakhon Province Campus, Sakon Nakhon 47000, Thailand; anusak.ke@ku.th; 4 Food Risk Hub, Research Unit of Chulalongkorn University, Bangkok 10330, Thailand

**Keywords:** peptidomics analysis, virulent peptides, *Streptococcus suis*, pathogenesis, virulence factor, zoonosis

## Abstract

**Simple Summary:**

The virulence factors and pathogenesis of *S. suis* are inconclusive. Here, the associated proteins, or their derived peptides, involved in the survival of *S. suis* when simulated with a blood environment are demonstrated. The results reveal the derived peptides or proteins of *S. suis* potentially serving as the putative virulence factors. Further studies based on our findings could be used to fulfill the knowledge gap of *S. suis* pathogenesis.

**Abstract:**

*Streptococcus suis* (*S. suis*) is a zoonotic pathogen causing severe streptococcal disease worldwide. *S. suis* infections in pigs and humans are frequently associated with the virulent *S. suis* serotype 2 (SS2). Though various virulence factors of *S. suis* have been proposed, most of them were not essentially accounted for in the experimental infections. In the present study, we compared the peptidomes of highly virulent SS2 and SS14 in humans, the swine causative serotypes SS7 and SS9, and the rarely reported serotypes SS25 and SS27, and they were cultured in a specified culture medium containing whole blood to simulate their natural host environment. LC-MS/MS could identify 22 unique peptides expressed in the six *S. suis* serotypes. Under the host-simulated environment, peptides from the ABC-type phosphate transport system (SSU05_1106) and 30S ribosomal protein S2 (*rps*B) were detected in the peptidome of virulent SS2 and SS14. Therefore, we suggest that these two proteins or their derived peptides might be involved in the survival of *S. suis* when simulated with a blood environment.

## 1. Introduction

*Streptococcus suis* is a zoonotic bacterium causing severe streptococcal infection in both humans and pigs. Among 35 serotypes classified by the immunogenics of their capsular polysaccharides [1], SS serotype 2 (SS2) is the most prevalent, followed by SS14, the causative agent of *S. suis* zoonotic infection in humans and pigs worldwide [2,3]. For swine infections, SS7 and SS9 are associated with the clinical swine cases in the European region while SS3 is usually disseminated in North America and Asia [2]. The clinical symptoms of *S. suis* infection range from a common bacterial infection to streptococcal toxic shock-like syndrome (TSST) [1,4]. Although some virulence factors of *S. suis* such as capsular polysaccharide (CPS), suilysin, muramidase-released protein (MRP), and extracellular factors have been described [5,6,7], a thorough list of virulence factors of *S. suis* particularly from some other serotypes also needs to be explored. Proteomic and peptidomic techniques have been widely adopted and used to investigate the microbial proteomes for the virulence factor, diagnostic marker, and vaccine targets [8,9]. In the present study, a high-performance liquid chromatography-mass spectrometry/mass spectrometry method (LC-MS/MS) was used to determine the peptidomes of the highly virulent to humans SS2 and SS14, swine causative serotypes SS7 and SS9, and rarely reported serotypes SS25 and SS27 cultured in a specified culture medium containing whole blood, simulating their natural host environment. This study aimed to explore some putative virulence factors involved in the survival of *S. suis* in the blood environment.

## 2. Methods

### 2.1. Bacterial Strains 

Six reference *S. suis* serotypes, originating from diseased pigs, were included in the present study. The virulent S. *suis* to humans were SS2 (ATCC 700794) and SS14 (13730) and the non-virulent *S. suis* to humans were SS7 (8074), SS9 (22083), SS25 (89-3576-3), and SS27 (89-5259). *S. suis* bacteria were cultured on Columbia blood agar (Difco Laboratories, Detroit, MI, USA) with 5% (*v*/*v*) sheep’s blood at 37 °C in anaerobic conditions using a GasPak Anaerobic System (Mitsubishi Gas Chemical Co., Inc., Tokyo, Japan) for 24 h. Sequencing of the 16S rRNA gene was performed to validate their strains [10]. GenBank accession numbers of the 16S rRNA gene of SS2, SS14, SS7, SS9, SS25 and SS27 are LS483418.1, AF009489.1, AF009482.1, AF009484.1, AF009500.1 and AF009502.1, respectively. The bacterial colonies were then cultured in a Todd–Hewitt broth (THB) (Difco Laboratories, Detroit, MI, USA) and preserved with 20% glycerol at −80 °C for further study.

### 2.2. Preparation of Peptidome

SS2, SS14, SS7, SS9, SS25, and SS27 were independently cultured in THB at 37 °C for 18 h. A mixture of 200 µL of cultured bacterium (≅10^7^ CFU/mL) of the *S. suis* serotype and 800 µL of fresh whole sheep’s blood (red blood cells ≅10^6^ cells/mL) was incubated at 37 °C for 30 min. The results of an MTT (3-[4,5-dimethylthiazol-2-yl]-2,5-diphenyltetrazolium bromide) assay [11] revealed that after 30 min of incubation time with whole blood, the optical density at 570 nm significantly rose (*p* < 0.01), representing the proliferation of *S. suis* viable cells (data not shown). We then assumed that virulence factors were triggered as part of the *S. suis* pathogenicity during the bacterial colonization in the host in as soon as 30 min of exposure to the blood culture medium. This procedure was repeated five times for each *S. suis* serotype. The bacterial peptides were extracted from the *S. suis* bacterial suspension according to a previous study [12]. Briefly, after centrifugation at 300 g for 5 min, 5% (*v*/*v*) trifluoroacetic acid (TFA) in absolute acetonitrile (ACN) was added to the pellet and the suspension was dissolved by gentle vortexing. The samples were dried to remove ACN and resuspended with 0.1% (*v*/*v*) formic acid. A Lowry assay was used to determine peptide concentration [13].

### 2.3. LC-MS/MS

An HCTUltra PTM Discovery System (Bruker Daltonics Ltd., Bremen, Germany) coupled with an UltiMate 3000 LC System (Dionex Ltd., Camberley, UK) was used to analyze peptides in each sample. The peptide samples were separated on a nanocolumn (Acclaim PepMap 100 column 75 um × 5 cm) using reversed-phase high-performance liquid chromatography. Two eluents were used. Eluent A was 0.1% formic acid and eluent B was 80% ACN in water containing 0.1% formic acid. A 5–55% eluent B gradient was used to elute peptides at a constant flow rate of 0.30 μL/min for 30 min. Electrospray ionization was carried at 1.6 kV using the CaptiveSpray. Nitrogen, a drying gas, was applied with a flow rate of about 50 L/h. Collision-induced-dissociation product ion mass spectra were achieved using nitrogen gas as the collision gas. Mass spectra (MS) and MS/MS spectra were collected in positive-ion mode at 2 Hz over the (*m*/*z*) range 150–2200. The collision energy was adjusted to 10 eV as a function of the *m*/*z* value. The LC-MS analysis of each sample was performed in triplicate. 

### 2.4. Peptidomic Data Analysis

DeCyder MS Differential Analysis Software (DeCyderMS, GE Healthcare) was used to quantitate peptides from MS/MS signal data. ANOVA statistical analysis, incorporated into the DeCyder MS Differential Analysis Software was used to analyze significantly different peptide peaks. The analyzed MS/MS data were submitted to identify proteins against the Uniprot database using the Mascot software (Matrix Science, London, UK). The parameters for the database search were taxonomy (*Streptococcus suis*), enzyme (NoCleave), variable modifications (oxidation of methionine residues), mass values (monoisotopic), protein mass (unrestricted), peptide mass tolerance (1.2 Da), fragment mass tolerance (±0.6 Da), peptide charge state (1+, 2+ and 3+), and missed cleavages (3). The MS/MS spectra were manually inspected for the full sequence of the main fragmentation series by Mascot. All differentially expressed peptides were analyzed for their intersections among the different sample groups using jvenn [14]. Information about particular proteins was used in the annotation by UniProtKB/Swiss-Prot entries (http://www.uniprot.org/ accessed on 29 July 2020). The relationships between identified proteins (from *S. suis*) and other interesting molecules were investigated using STITCH 5.0 (http://stitch.embl.de/ accessed on 24 August 2020) [15].

## 3. Results

### Peptidomic Analysis by LC-MS/MS 

**Figure 1:**
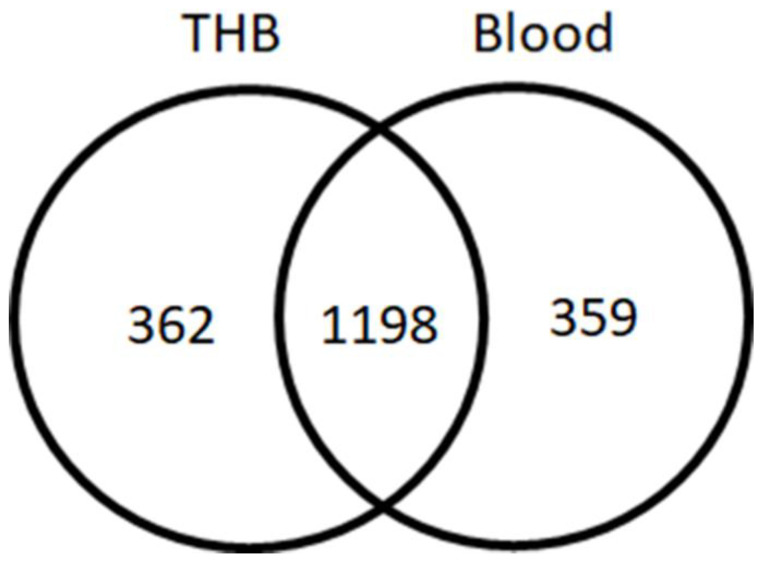
Venn diagram showing the number of proteins in SS2 cultivated in the THB medium with and without whole blood.

The different expressions of *S. suis* peptides cultured using the THB medium and the THB medium containing whole blood were determined using LC-MS/MS. A total of 1919 peptides were identified in the peptidome of SS2. All 1919 peptides were significantly identified using the DeCyder MS Differential Analysis software (*p* < 0.05). The distributions of peptides of SS2 cultivated in certain conditions were shown in Figure 1. Only peptides found in SS2 grown in the THB medium supplemented with the whole blood (359 peptides) were used for further investigation (Figure 1). Likewise, peptides identified in SS14, SS7, SS9, SS25, and SS27 were included for further investigation. The complete list of peptides for all six serotypes was provided in the Supplementary Materials.

Overall, 22 peptides derived from SS2, SS14, SS7, SS9, SS25, and SS27 proteins with known functions, matched in the STITCH database, were selected and are shown in Table 1. In particular, three peptides were identified only in virulent *S. suis*. Interestingly, out of three peptides, two of them were consistently detected in virulent *S. suis* serotype 2 and/or serotype 14 including the ABC-type phosphate transport system (SSU05_1106) and 30S ribosomal protein S2 (rpsB), whereas 12 peptides were expressed only in non-virulent *S. suis* SS7, SS9, SS25, and SS27 (Table 2). They were transcriptional regulator (SSU05_2039), α-1,4 glucan phosphorylase, PTS system ascorbate-specific transporter subunit IIC (SSU05_2063), branched-chain amino acid permease (SSU05_0780), 10 kDa chaperonin (GroS), low-temperature requirement A (SSU05_0642), Cps2J (cps2J), truncated MRP, hypothetical protein (SSU05_1457), hypothetical protein (SSU05_0461), hypothetical protein (SSU05_1869) and a hypothetical protein (SSU05_0141). 

A peptide 5′-nucleotidase/2′,3′-cyclic phosphodiesterase and related esterases (SSU05_2116), associated with nucleotide catabolic activity, was detected in SS2, SS14, and SS9, while the transcriptional regulator protein (SSU05_2039) was found in SS7 and SS25. Three peptides related to carbohydrate metabolism were identified, including; K01223 6-phospho-beta-glucosidase (SSU05_1489) in SS2, SS14, and SS9, α-1,4 glucan phosphorylase in SS9 and SS25, and 4-α-glucanotransferase (SSU05_0392) in SS14. Moreover, a peptide of fatty acid biosynthesis protein, 3-oxoacyl-[acyl-carrier-protein] reductase (SSU05_1803) was also identified in SS2, SS14, SS7, and SS27.

Peptides involved in the membrane of the bacteria were also detected, consisting of a derived peptide of the PTS system ascorbate-specific transporter subunit IIC (SSU05_2063) in SS7 and SS9, ABC transporter permease (SSU05_1254) in SS14 and SS7, and branched-chain amino acid permease (SSU05_0780) in SS7.

Other peptides were guanylate kinase (gmk) expressed in SS2, SS14, SS9, SS25, and SS27, and tetracycline resistance protein (TetM) was identified in SS14, SS25, and SS27. Peptide co-chaperonin GroS was found in SS7. A putative low-temperature requirement A protein (SSU05_0642) was found in SS7. Furthermore, Cps2J was identified in SS25, and a truncated MRP was found in SS7.

Various peptides belonged to a hypothetical group, such as SSU05_1457 hypothetical protein in SS25, SSU05_0461 hypothetical protein in SS27, SSU05_1869 hypothetical protein in SS25, and SS27, SSU05_0141 hypothetical protein in SS25, and SSU05_1991 hypothetical protein in SS2, SS7, SS25, and SS27.

## 4. Discussion

*S. suis* pathogenesis is triggered by exposure to a certain condition in the host environment [16]. In the present study, six *S. suis* serotypes were simulated under the human cardiovascular system and they interacted with erythrocytes, leukocytes, and other blood components particularly during bacterial septicemia (bacteremia). A large number of peptides were identified in the peptidome of *S. suis*. Among peptides expressed by reacting with the whole blood, only 22 unique peptides derived from *S. suis* proteins with known functions, matched in the STITCH database, were selected (Table 1). Interestingly, derived peptides of the ABC-type phosphate transport system and 30S ribosomal protein S2 were simultaneously co-expressed in the highly virulent SS2 and virulent SS14 cultured under the host-simulated condition. Notably, the ABC transporter protein has been reported in the pathogenicity of *S. pneumoniae* [17], while 4-α-glucanotransferase (*malQ* gene), responsible for starch metabolism in *S. mutans,* was expressed uniquely in the virulent SS14 [18]. Therefore, derived peptides of the ABC-type phosphate transport system, 30S ribosomal protein S2 and 4-α-glucanotransferase might serve as specific virulence factors of SS2 and SS14.

The association of 5′-nucleotidase/2′,3′-cyclic phosphodiesterase and related esterases (SSU05_0456) with *S. suis* has been rarely mentioned to the best of our knowledge. However, 5′-nucleotidase has been considered as a virulence factor in many pathogens including *S. suis* [19,20,21,22], because this enzyme is commonly involved in adenosine synthesis. Adenosine, an immunomodulatory molecule, reduces the phagocytic activity of macrophages and also impairs the neutrophil degranulation thus decreasing the inflammatory responses of the host immune system [19]. A previous study by Liu et al., (2014) demonstrated that 5′-nucleotidase is an essential factor for *S. suis* escaping from the host’s innate immunity as the enzyme-deficient mutant strain of *S. suis* lost its infectivity in the pig model [20]. Thus, our result was in concordance with Liu et al., (2014) as a derived peptide of 5′-nucleotidase was found in the virulent SS2 and SS14 reacting with the blood in the host-simulated environment. This emphasized the significance of the survival proteins and/or peptides initially expressed to tackle the host-simulated immunity.

The α-1,4 glucan phosphorylase, K01223 6-phospho-β-glucosidase [EC-3.2.1.86] and the 4-α-glucanotransferase associated with carbohydrate metabolism were exhibited in SS9 and SS25; SS2, SS14, and SS9; and SS14, respectively. These results are compatible with a study by Zhang et al., (2014) that identified 6-phospho-β-glucosidase in two Chinese isolates of virulent SS2 [23]. Unfortunately to the best of our knowledge, the direct association of these three enzymes with *S. suis* is not evident. However, the carbohydrate availability is positively correlated with the virulence of *S. suis* using the host cell invasion i.e., the adequate carbohydrate supply upregulates the virulence gene regulation of virulent *S. suis* as described previously in a transcriptomic study [24].

The biosynthesis of fatty acids is important for the pathogenicity and adaptation of bacteria in the host environment since fatty acids serve as an energy source and a material for cell-wall synthesis. In addition, the pattern of the lipid cell walls also acts as a factor modulating the immune responses of the host [25,26]. In the present study, a derived peptide of 3-oxoacyl-[acyl-carrier-protein] reductase responsible for the fatty acid biosynthesis was identified in SS2, SS14, SS7, and SS27. The previous studies demonstrated that this enzyme was associated with the fatty acid biosynthesis of virulent SS2 [27,28]. Thus, we could assume that this fatty acid biosynthesis-associated enzyme is significant for *S. suis* adaptation in the host-simulated environment. 

**Figure 2:**
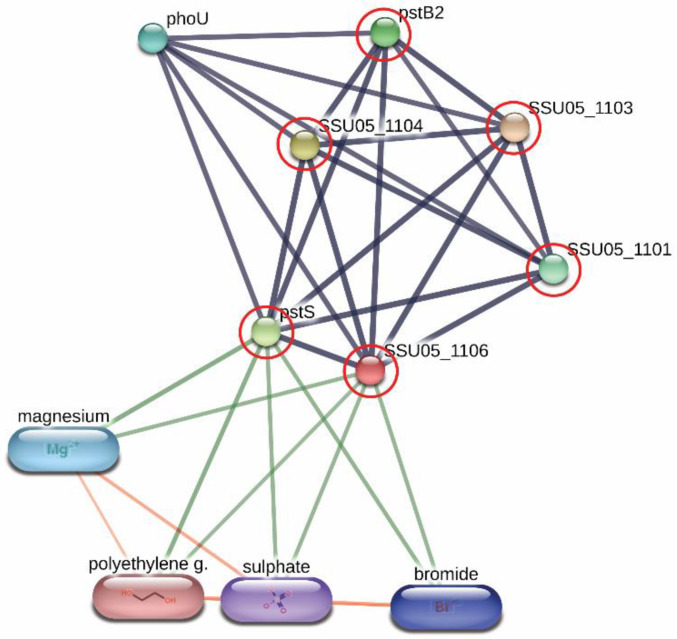
Relationship between ABC-type phosphate transport system (SSU05_1106) and other phosphate ABC transporters; SSU05_1101, SSU05_1103, SSU05_1104, pstS and pstB2 involved in phosphate import from STITCH 5.0 software. The grey thick lines represent the highest edge confidence scores (0.900) indicating the high strength of the protein interactions at the functional level.

Derived peptides of the ABC transporter permease and the ABC-type phosphate transport system were found in the peptidomes of SS14 and SS7, and SS2 and SS14, respectively. Generally, ABC transporter proteins play an important role in the transportation of various substrates including nutrients for the biosynthesis of the bacterial cell. Some of the ABC transporters serve as efflux pumps. Previous studies reported that the increased expression of an ABC efflux pump was related to fluoroquinolones resistance in *S. pneumoniae* and *S. suis* [29,30]. As shown in Figure 2, the networks of protein–protein interactions analyzed by the STITCH program, version 5.0, demonstrated high associations of the ABC-type phosphate transport system (SSU05_1106) with other phosphate ABC transporters (edge confidence score 0.900), responsible for phosphate import. Furthermore, these proteins are associated with the biofilm formation of SS2 [31] and are required for the disease pathogenesis of *S. pneumoniae* [17]. The branched-chain amino acid permease, one of the ABC transporter proteins, is involved in the transport of leucine, isoleucine, and valine [32]. A previous report also suggested that the branched-chain amino acid permease was related to the pathogenicity of some bacteria [17]. A certain role for the ABC transporter as a virulence factor of bacteria is still not clear. However, the presence of ABC transporters in the virulent to humans SS2, SS14, and non-virulent to humans SS7 indicated that the translocation of substrates particularly phosphate and some amino acids (leucine, isoleucine, and valine) using these ABC transporter proteins is perhaps required for the survival and then the pathogenesis (pathway) of virulent *S. suis* in the host-simulated environment.

Guanylate kinase phosphorylates the guanosine monophosphate to guanosine diphosphate, a precursor of the guanosine triphosphate required for the transcription of RNA synthesis. This enzyme was inhibited when the bacteria were forced to survive in the amino-acid restricted condition [33]. In the present study with adequate nutrients in the host-simulated environment, a peptide of this enzyme was identified in SS2, SS14, SS9, SS25, and SS27. This finding was in line with a previous report indicating that this enzyme is conserved and adaptively expressed depending on the nutrient availability [33].

The tetracycline-resistance gene, *tet*M, encoded TetM protein responsible for the inhibitory effect of tetracycline, is distributed among virulent *S. suis.* [34,35,36]. An outbreak report in China demonstrated that the tetracycline-resistance strains were associated with *S. suis* infections in both pigs and humans [34]. Our results revealed the expression of the TetM protein in SS14, SS25, and SS27 cultured under a host-simulated environment. This finding is in concordance with a previous report indicating that *S. suis* carried and spread the *tet*M gene among *S. suis* strains [34].

The association of *S. suis* and 10 kDa chaperonin is rarely reported. This protein is specified as a protein folding facilitator and also a virulence factor of bone resorption in *Mycobacterium tuberculosis* infection [37]. In the present study, a peptide derived from 10 kDa chaperonin was detected in the peptidome of SS7. 

So far, the CPS of *S. suis* has been mentioned as an important virulence factor of *S. suis* [7]. The *cps* gene cluster facilitates the regulation of CPS synthesis. The Cps2J is essential for CPS biosynthesis because the deletion mutant of ∆*cps*2J results in the imperfection of CPS [38]. The presence of Cps2J only in SS25 might explain why the bacterium was likely to synthesize CPS to protect itself from phagocytosis by the host immune cells [7]. Cps2J was identified in SS14 and SS25 while Cps2A and Cps2C were identified in SS2, SS14, SS9, and SS25 (data not shown). 

Previously, MRP has been considered as a virulent marker of *S. suis* [39]. The mutant strain of *S. suis* demonstrated its virulence even when the MRP protein was deficient. Thus, MRP is more likely to be the virulent maker than the virulence factor. This protein was proposed to be coincidentally expressed together with the true virulence factor [16]. In this study, we identified a peptide derived from truncated MRP only in SS7 under the host-simulated environment. The limited existence of this protein might be explained by the fact that MRP is a cell-wall anchored protein and is released into the supernatant part of the culture medium. Our peptide preparation method did not precipitate proteins in the supernatant thus decreasing the chance of capturing MRP in *S. suis* cells. A previous study focusing on proteomic analysis of SS2 human isolates also reported no expression of MRP either [40].

Other unique proteins identified in this study were putative low-temperature requirement A protein (SSU05_0642), transcriptional regulator (SSU05_2039), and 30S ribosomal protein S2(rpsB). Although the related information regarding their virulence is not available, the expression of 30S ribosomal protein S2 should not be neglected, since these proteins were exhibited only in virulent SS2 and SS14 culture under the host-simulated environment. The role of these proteins in conjunction with the pathogenesis of virulent *S. suis* deserves further study.

Among five hypothetical proteins presented in this study (Table 1), SSU05_1869 hypothetical protein was previously mentioned as a putative ABC transporter protein [41] and SSU05_1991 hypothetical protein was described as possibly associated with bacterial cell growth and division in *S. suis* serotype 2 [42]. In the present study, SSU05_1991 hypothetical protein was also exhibited in SS2 and also in SS7, SS25, and SS27. When the functions or subcellular locations of the other three hypothetical proteins SSU05_1457, SSU05_0461, and SSU05_0141 are identified, their roles regarding the pathogenesis of virulent *S. suis* will be disclosed.

## 5. Conclusions

*S. suis* expressed its virulence factor in the form of proteins or peptides to survive in the host. Our study demonstrated various derived peptides expressed in *S. suis* SS2, SS14, SS7, SS9, SS25, and SS27 reacting with whole blood. Most unique proteins were associated with the transcriptional process and cellular metabolic processes, thus indicating the adaptation of the bacterial cell during the initial period of incubation time in the host-simulated environment. The peptides of the ABC-type phosphate transport system (SSU05_1106) and 30S ribosomal protein S2 (rpsB) were solely expressed in virulent to humans SS2 and SS14 under the host-simulated environment and are proposed as putative virulence factors potentially involved in *S. suis* pathogenesis. The results of this study apply to the diagnostic marker and vaccine targets [8,9]. However, our results only demonstrated the identified peptides by in vitro simulating environment. Further study by in vivo and gene knockout should be conducted to verify our peptidomic findings.

## Figures and Tables

**Table 1:** Identified peptides derived from *S. suis* proteins with known function cultured under the host simulated environment.

Gene Locus	Annotated Protein Function (Amino-Acid Length)	Function or Subcellular Location
SSU05_2116	5′-nucleotidase/2′,3′-cyclic phosphodiesterase and related esterases (448)	nucleotide catabolic activity
SSU05_2039	transcriptional regulator (149)	DNA-binding transcription factor activity
N/A	α-1,4 glucan phosphorylase (755)	carbohydrate metabolism
SSU05_1489	K01223 6-phospho-beta-glucosidase [EC-3.2.1.86] (474)	carbohydrate metabolism
SSU05_0392	4-α-glucanotransferase (364)	carbohydrate metabolism
SSU05_1803	3-oxoacyl-[acyl-carrier-protein] reductase (244)	fatty acid biosynthesis
SSU05_2063	PTS system ascorbate-specific transporter subunit IIC (482)	cell membrane/membrane
SSU05_1254	ABC transporter permease (197)	transmembrane transporter
SSU05_1106	ABC-type phosphate transport system (83)	periplasmic component
SSU05_0780	branched-chain amino acid permease (188)	cell membrane/membrane
*gmk*	guanylate kinase (216)	GMP recycling
*tet*M	TetM (639)	response to antibiotic
*gro*S	10 kDa chaperonin (102)	ATPase activity
SSU05_0642	putative low-temperature requirement A (371)	transmembrane protein
*cps*2J	Cps2J (332)	capsular biosynthesis
*mrp*	truncated MRP (1261)	secreted protein
*rps*B	30S ribosomal protein S2 (258)	ribosomal protein
SSU05_1457	hypothetical protein (91)	
SSU05_0461	hypothetical protein (567)	
SSU05_1869	hypothetical protein (178)	
SSU05_0141	hypothetical protein (318)	
SSU05_1991	hypothetical protein (412)	

**Table 2:** Distribution of 22 identified peptides derived from *S. suis* proteins of virulent vs. non-virulent *S. suis* to human cultured under host simulated environment.

Identified Protein	*S. suis* Serotype					
	Virulent		Non-Virulent			
	2	14	7	9	25	27
5′-nucleotidase/2′,3′-cyclic phosphodiesterase and related esterases	+	+		+		
transcriptional regulator			+		+	
α-1,4 glucan phosphorylase				+	+	
K01223 6-phospho-beta-glucosidase [EC-3.2.1.86]	+	+		+		
4-α-glucanotransferase		+				
3-oxoacyl-[acyl-carrier-protein] reductase	+	+	+			+
PTS system ascorbate-specific transporter subunit IIC			+	+		
ABC transporter permease		+	+			
ABC-type phosphate transport system	+	+				
branched-chain amino acid permease			+			
guanylate kinase	+	+		+	+	+
TetM		+			+	+
10 kDa chaperonin			+			
putative low-temperature requirement A			+			
Cps2J					+	
truncated MRP			+			
30S ribosomal protein S2	+	+				
SSU05_1457 hypothetical protein					+	
SSU05_0461 hypothetical protein						+
SSU05_1869 hypothetical protein					+	+
SSU05_0141 hypothetical protein					+	
SSU05_1991 hypothetical protein	+		+		+	+
Sum	7	9	9	5	9	6

## Data Availability

Roytrakul, Sittiruk; Nuanualsuwan, Suphachai; Chaiden, Chadaporn; Phaonakrop, Narumon; Jaresitthikunchai, Janthima (2021): Membrane_Peptidome_mzXML.rar. figshare. Dataset. https://doi.org/10.6084/m9.figshare.14794899.v1 (accessed on 17 June 2021).

## Supplementary Materials

The following are available online at https://www.mdpi.com/article/10.3390/ani11092480/s1. Table S1: The list of peptides of SS2; Table S2: The list of peptides of SS14; Table S3: The list of peptides of SS7; Table S4: The list of peptides of SS9; Table S5: The list of peptides of SS25; Table S6: The list of peptides of SS27.
